# Supplementary material for: Wilson Disease: Intersecting DNA Methylation and Histone Acetylation Regulation of Gene Expression in a Mouse Model of Hepatic Copper Accumulation
Source: Cell Mol Gastroenterol Hepatol. 2021 Jun 4;12(4):1457–77. doi: 10.1016/j.jcmgh.2021.05.020 (PMC8487080; doi:10.1016/j.jcmgh.2021.05.020)
Supplement: Supplementary Data S1 [file mmc1.docx]

**Supplementary Information for**

**Wilson disease: intersecting DNA methylation and histone acetylation in a mouse model of hepatic copper accumulation**

Gaurav V. Sarode^1^, Kari Neier^2^, Noreene M. Shibata^1^, Yuanjun Shen^3^, Dmitry A Goncharov^3^, Elena A. Goncharova^3^, Tagreed A. Mazi^4,5^, Nikhil Joshi^6^, Matthew L. Settles^6^, Janine M. LaSalle^2^*, Valentina Medici^1^*

* Authors share co-senior authorship

Corresponding author:

Valentina Medici, M.D., F.A.A.S.L.D.

Professor

University of California, Davis

Department of Internal Medicine, Division of Gastroenterology and Hepatology

4150 V Street, PSSB Suite 3500

Sacramento, California 95817

vmedici@ucdavis.edu

**Table of Contents**

| Section | Page |
| --- | --- |
| Supplementary Figures | 2 |
| Supplementary Tables | 8 |

**Figure S1: Age- and dose-dependent immunoblots of HDAC4 and HDAC5 in mouse and HepG2 models of WD.**


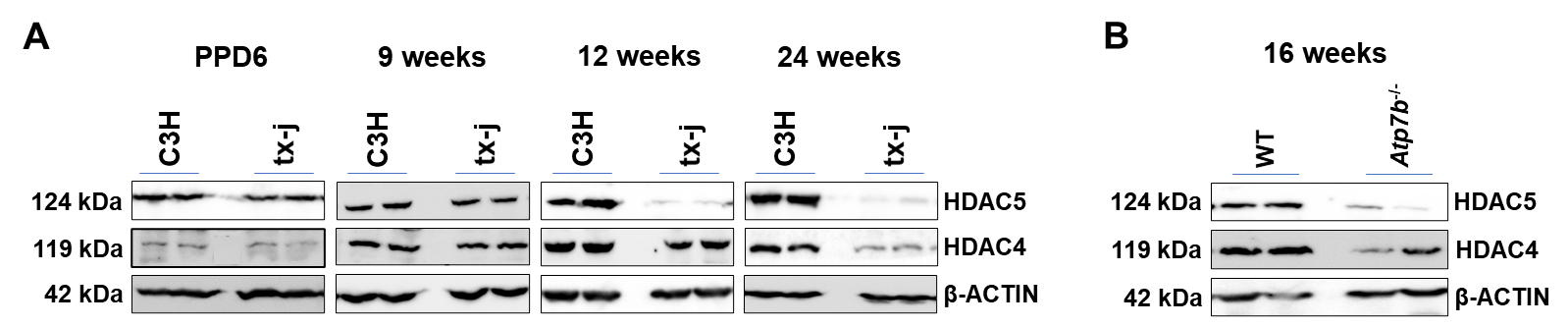


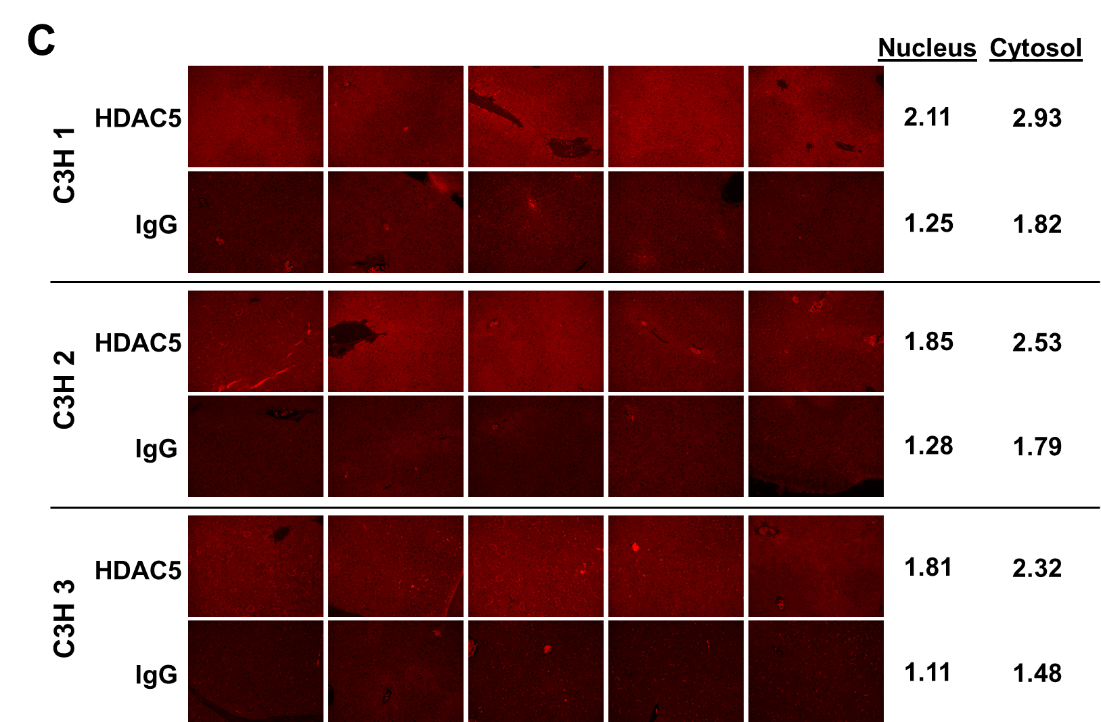


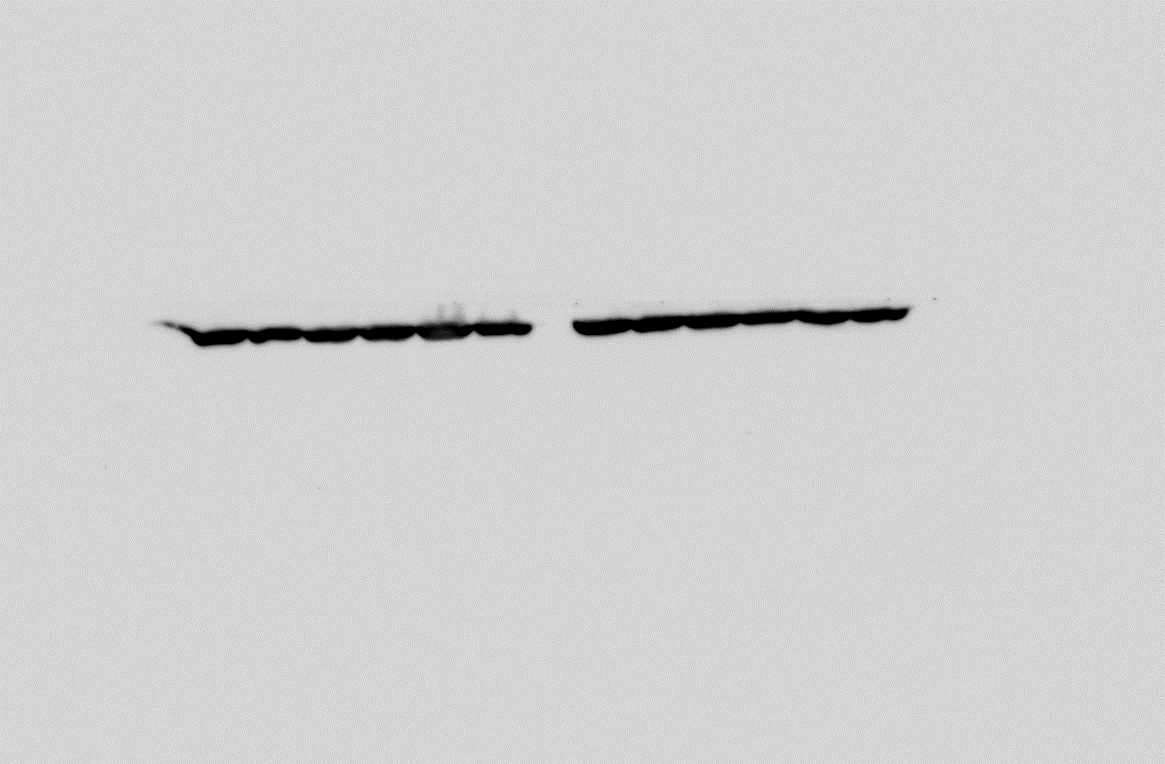

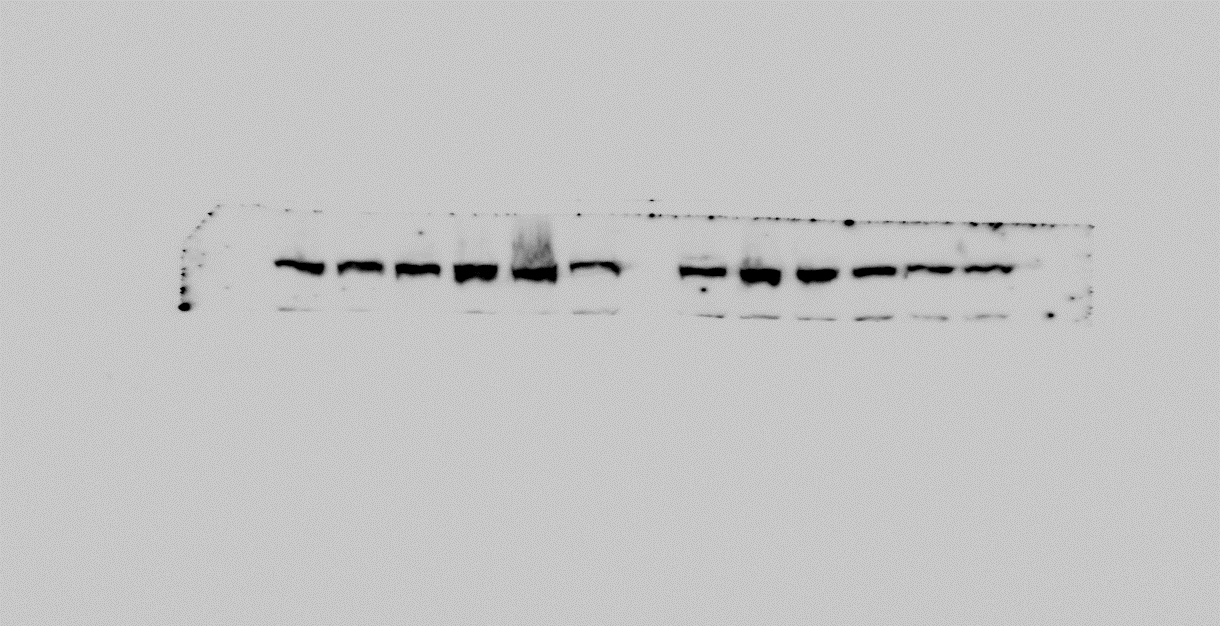


**42 kDa**

**124 kDa**

**HDAC5**

**β-ACTIN**

**µM 0 5 10 25**

**HepG2 cells: IPN treatment**

**D**


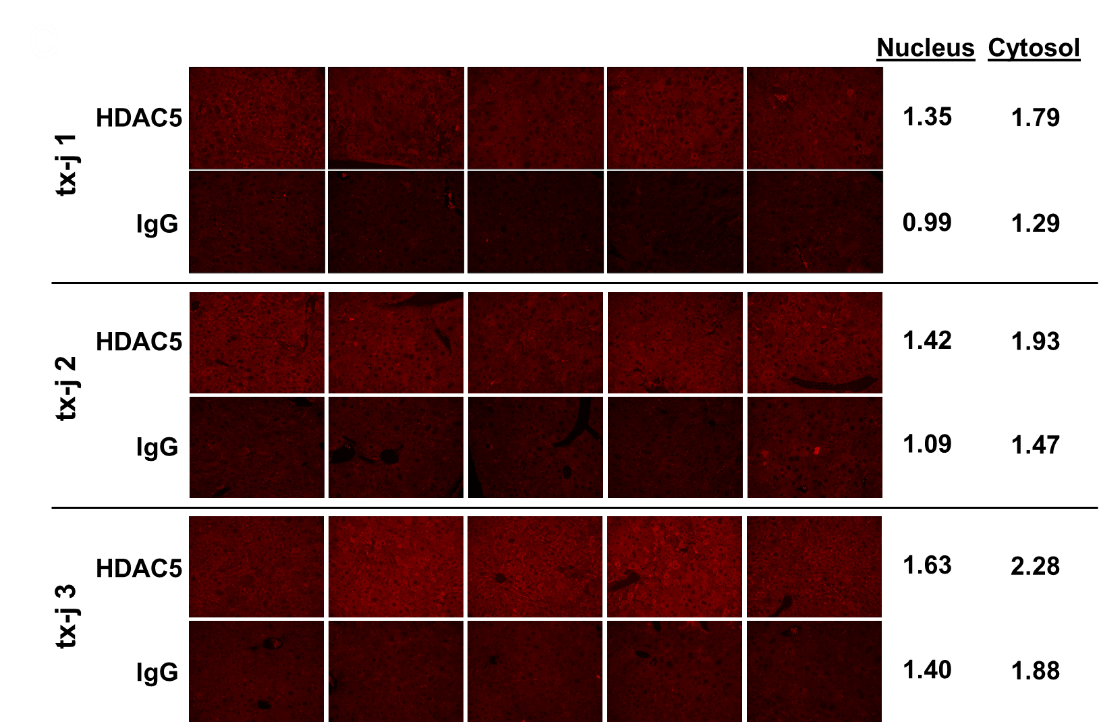


A: Total protein liver lysate for histone deacetylase (HDAC) 4 and HDAC5 protein expression in tx-j mice compared to C3H control at post-partum day 6 (PPD6; C3H n=4M/4F, tx-j n=5M/4F), 9 weeks (C3H n=4M/3F, tx-j n=4M/4F), 12 weeks (C3H n=4M/4F, tx-j n=4M/3F), and 24 weeks (C3H n=10M/12F, tx-j n=11M/11F). Immunoblot images show 2 representative samples per group per time point. B: Total protein liver lysate for HDAC4 and HDAC5 protein expression in 16-week old *Atp7b*^-/-^ (n=7M/4F) mice and wild-type (WT, n=6M/5F). Immunoblot images show 2 representative samples per group. C: Immunoblot of HepG2 cell lysates treated with 50µM of CuSO_4_ for 24 hours followed by 24-hour treatment with isoproterenol (IPN, 0-25µM, n=3), a HDAC5 activator. β-ACTIN was used as loading control in all immunoblots.

**Figure S2: Immunoblots of AMPKα signaling in 24-week old tx-j mice and AICAR treatment in HepG2 cells.**


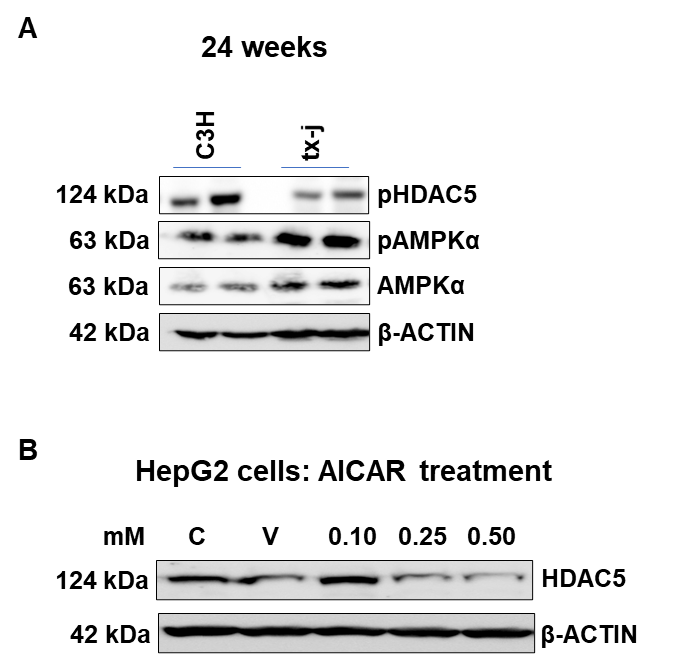


A: Total protein liver lysates of total AMPKα, phosphorylated AMPKα (pAMPKα), and phosphorylated HDAC5 (pHDAC5) obtained from 24-week old C3H (n=3M/3F) and tx-j (n=3M/2F). Immunoblot images show 2 representative samples per group. B: HDAC5 immunoblot of HepG2 cell lysates treated with 50 µM CuSO_4_ for 24 hours followed by 5-aminoimidazole-4-carboxamide-1-β-D-ribofuranoside (AICAR) treatment (0-0.5 mM; n=3), an AMPK activator, for 24 hours. C=control, V=treated with vehicle (DMSO) only.

**Figure S3: Immunoblots of HDACs and H3 marks in response to PCA and choline.**


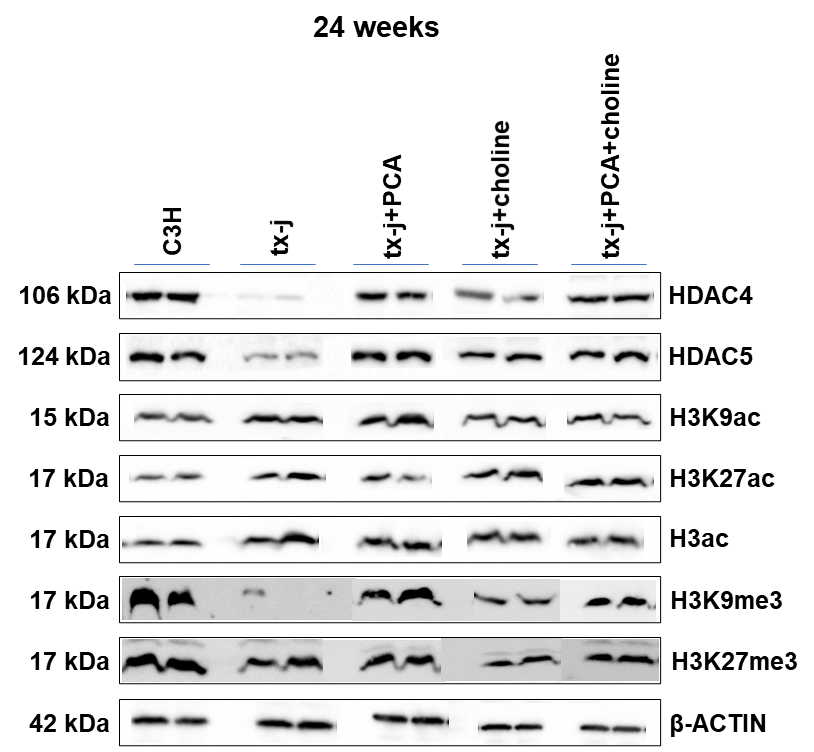


Immunoblots of total protein liver lysates from 24-week old C3H (n=10M/12F) and tx-j mice (n=11M/11F), and tx-j mice treated with PCA (n=11M/10F), choline (n=8M/13F), and PCA+choline (n=10M/11F). Immunoblot images show 2 representative samples per group.

**Figure S4: ChIP-seq and RNA-seq volcano plots.**

Volcano plots showing the separation of differentially regulated genes (p<0.05) in both ChIP-seq (left) and RNA-seq (right) comparing livers of 3M/3F 24-week old C3H vs. tx-j mice. Data are represented as -log p-value vs. log2 fold change.

**Figure S5: HDAC5, PPARγ, and PPARα immunoblots in response to dietary modulation.**


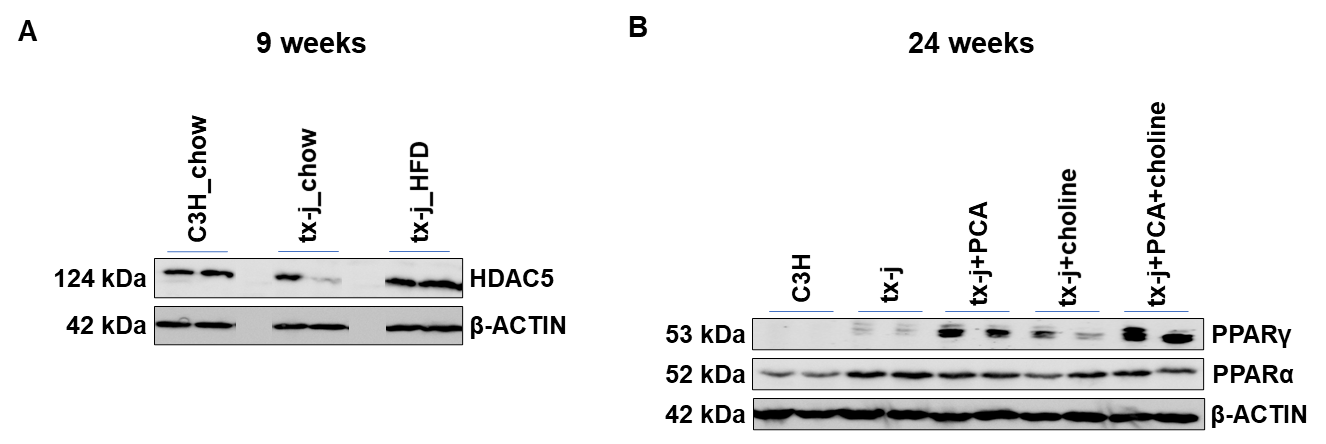


A: HDAC5 protein expression in total protein liver lysate from 9-week mice on chow (C3H n=4M/3F, tx-j n=4M/4F) and fed a 60% kcal fat diet (HFD, n=4M/4F each) for 8 days. B: Immunoblots of PPARγ and PPARα in total protein liver lysate of 24-week old C3H, tx-j, tx-j+PCA, tx-j+choline, and tx-j+PCA+choline (n=3M/3F per group). Immunoblot images show 2 representative samples per group.

**Table S13: Primers for qPCR analyses.**

| *Gene* | Full name | Primer | Sequence 5' to 3' | Accession | Exon-exon overlap | % Primer efficiency |
| --- | --- | --- | --- | --- | --- | --- |
| *Aebp1* | AE binding protein1 | F | CCTCGCTCTGGGACTTTCAA | NM_009636.3 | No | 99.47 |
|  |  | R | CGGGGTAGCTCACTCTCGTG |  | Yes |  |
| *Aebp2* | AE binding protein2 | F | AGCTCGCCATGTACCCACAC | NM_001005605.2 | No | 94.76 |
|  |  | R | CCTCCGCTTGTTCATTCCAG |  | No |  |
| *Ampka1* | Protein kinase, AMP-activated, alpha 1 catalytic subunit | F | GAACGCATTTGGAGGACATGA | NM_001013367 | No | 99.35 |
|  |  | R | AGCCCCGAACAAAAAGAAGC |  | No |  |
| *Atf4* | Activating transcription factor 4 | F | GGAAGCCTGACTCTGCTGCT | NM_001287180.1 | No | 95.64 |
|  |  | R | CTCCGGGCTCATACAGATGC |  | No |  |
| *Bcl2* | B cell leukemia/lymphoma 2 | F | AGGGCCTGAACTTGCGTGAA | NM_009741.5 | No | 102 |
|  |  | R | AGACAAGCGAGCTGGACAGG |  | No |  |
| *Cd36* | CD36 molecule | F | TGGGACCATTGGTGATGAAA | NM_001159555 | No | 99.65 |
|  |  | R | CCAATCCCAAGTAAGGCCATC |  | No |  |
| *Chrebp* | Carbohydrate response element binding protein (ChREBP | F | CCCCTCAGACACCCACATCTTC | NM_021455.5 | Yes | 95.2 |
|  |  | R | AGACGAGGCTGGAGATGGCA |  | No |  |
| *Fxr* | Farnesoid X receptor | F | CGAATGCCCGCTGAGACTGG | NM_009108.2 | No | 94.23 |
|  |  | R | CCCCTTTTATTCTGCCTGCCGA |  | No |  |
| *Gapdh* | Glyceraldehyde-3-phosphate dehydrogenase | F | GAAGCTTGTCATCAACGGGAAG | NM_008084.2 | No | 102.1 |
|  |  | R | TTTGATGTTAGTGGGGTCTCGC |  | No |  |
| *Gpx3* | Glutathione peroxidase 3 | F | TGGCTTGGTCATTCTGGGCTT | NM_008161.4 | No | 99.64 |
|  |  | R | CCCCACCTGGTCGAACATACTT |  | Yes |  |
| *Hat1* | Histone acetyltransferase 1 | F | CAGCGGAAGATCCGTCCA | NM_026115 | No | 106.2 |
|  |  | R | GCCATATCTTCACTGAATCCTTGC |  | Yes |  |
| *Hmgb2* | HMG box domain containing 4 | F | AGGAGACAGAGGGCATGGTG | NM_178017.2 | Yes | 107.32 |
|  |  | R | GGACAGCCGTTCAGTTCAGG |  | No |  |
| *Hmox1* | Heme oxygenase 1 | F | ATGCCCCAGGATTTGTCTGA | NM_010442.2 | No | 96.99 |
|  |  | R | TCTGGACACCTGACCCTTCTG |  | No |  |
| *Hnf4a* | Hepatic nuclear factor 4, alpha | F | CGCCCCGGTTGACTCTTGAT | NM_008261.3 | No | 96.4 |
|  |  | R | TCCAGTCTCACAGCCCATTCCT |  | No |  |
| *Hnf6* | Hepatic nuclear factor 4 | F | GGGTTGGAGCTGAGCACTGT | NM_008262.3 | No | 99.62 |
|  |  | R | TGCTCGATGAGGACGATGAA |  | No |  |
| *Irf6* | Interferon regulatory factor 6 | F | GCTCATTGCCCACCAGAAAG | NM_016851.2 | No | 98.64 |
|  |  | R | ACTGGGATGACCTGGACCAA |  | Yes |  |
| *Klf4* | Kruppel-like factor 4 | F | CACAGGCGAGAAACCTTACCA | NM_010637.3 | Yes | 97.12 |
|  |  | R | CGCACTTCTGGCACTGAAAG |  | No |  |
| *Lpl* | lipoprotein lipase | F | AACCAAGTCTGGCCTCGAACT | NM_008509 | No | 101.2 |
|  |  | R | AAGCTCCCAGGACACAGGAA |  | No |  |
| *Mmp12* | matrix metallopeptidase 12 | F | TGGAATATGACCCCCTGTTC | NM_008605.3 | No | 105.2 |
|  |  | R | CCAGCAAGCACCCTTCACTA |  | No |  |
| *Pparα* | peroxisome proliferator-activated receptor alpha | F | CGATGCTGTCCTCCTTGATGA | NM_011144.6 | No | 98.5 |
|  |  | R | GAAGTCAAACTTGGGTTCCATGAT |  | No |  |
| *Pparγ* | Peroxisome proliferator activated receptor gamma | F | CCGAAGAACCATCCGATTGA | NM_011146.3 | No | 105.27 |
|  |  | R | GAGACATCCCCACAGCAAGG |  | No |  |
| *Smad4* | SMAD family member 4 | F | TTGCTGAGTCTTCCCTGCTGG | NM_008540.3 | No | 98.63 |
|  |  | R | AGCTCTGACTCTCCTCGGCA |  | No |  |
| *Sod2* | Superoxide dismutase 2, mitochondrial | F | CAAGCACAGCCTCCCAGACC | NM_013671.3 | No | 98.08 |
|  |  | R | TGGCGTTGAGGTTGTTCACGT |  | No |  |
| *Sox6* | SRY (sex determining region Y)-box 6 | F | GCGACACAATGTACTGCGTTTT | NM_009238.3 | No | 97.09 |
|  |  | R | TGCTATCACCATGCCATAGGAC |  | No |  |
| *Srebf1* | sterol regulatory element binding transcription factor 1 | F | CTGGCTTGGTGATGCTATGTTG | NM_011480.3 | No | 104.9 |
|  |  | R | GACCATCAAGGCCCCTCAA |  | No |  |
| *Stard6* | StAR-related lipid transfer (START) domain containing 6 | F | AAAATTGCCTGCATCGGTAA | NM_029019.4 | No | 104.07 |
|  |  | R | CACGTATGGGTGGAATTCTG |  | Yes |  |
| *Ucp2* | Uncoupling protein 2 (mitochondrial, proton carrier | F | CGTCATCGCCTCCCCTGTTG | NM_011671.5 | No | 98.88 |
|  |  | R | CCGGAGCATGGTAAGGGCAC |  | No |  |
